# Supplementary material for: EPI-SauriCas9-based mouse ovarian cancer models recapitulating pten deletion in patients
Source: Commun Biol. 2025 Dec 29;9:159. doi: 10.1038/s42003-025-09437-2 (PMC12873370; doi:10.1038/s42003-025-09437-2)
Supplement: Supplementary file 2 — Supplementary Information [file 42003_2025_9437_MOESM2_ESM.pdf]

Supplementary Files

EPI-SauriCas9-Based Mouse Ovarian Cancer Models Recapitulating PTEN Deletion in Patients

Wutao Chen[1,5,8], Pengju He[2,8], Ling Ding[6,8], Weihua Lou[1,8], Yishu Wang[7], Weiwei Shi[1,5], Zhangzhengyi Fan[2], Yumeng Sheng[2], Jing Luo[2], Zhixing Tan[2], You Wang[1,2,5,9], Wen Di[1,2,5,9], Xiaoping Ke[4,9], Bin Yu[2,3,9]

[1]Department of Obstetrics and Gynecology, Renji Hospital, School of Medicine, Shanghai Jiao Tong University, Shanghai 200127, China
[2]State Key Laboratory of Systems Medicine for Cancer, Renji Hospital, School of Medicine, Shanghai Jiao Tong University, Shanghai 200127, China
[3]Shanghai Key Laboratory for Cancer Systems Regulation and Clinical Translation (CSRCT), Shanghai 200127, China
[4]Department of Obstetrics and Gynecology, Yangpu Hospital, School of Medicine, Tongji University, Shanghai, 200090, China
[5]Shanghai Key Laboratory of Gynecologic Oncology, Renji Hospital, School of Medicine, Shanghai Jiao Tong University, Shanghai 200127, China
[6]Traditional Chinese Medicine Department, Renji Hospital, School of Medicine, Shanghai Jiao Tong University, Shanghai 200127, China
[7]Department of Neurology, Renji Hospital, School of Medicine, Shanghai Jiao Tong University, Shanghai 200127, China
[8]These authors contributed equally
[9]These authors jointly supervised this work
Correspondence email: wanghh0163@163.com (Y.W.), diwen163@163.com (W.D.), Xiaoping.Ke@tongji.edu.cn (X.P.K), yubinrenji@outlook.com (B.Y.)

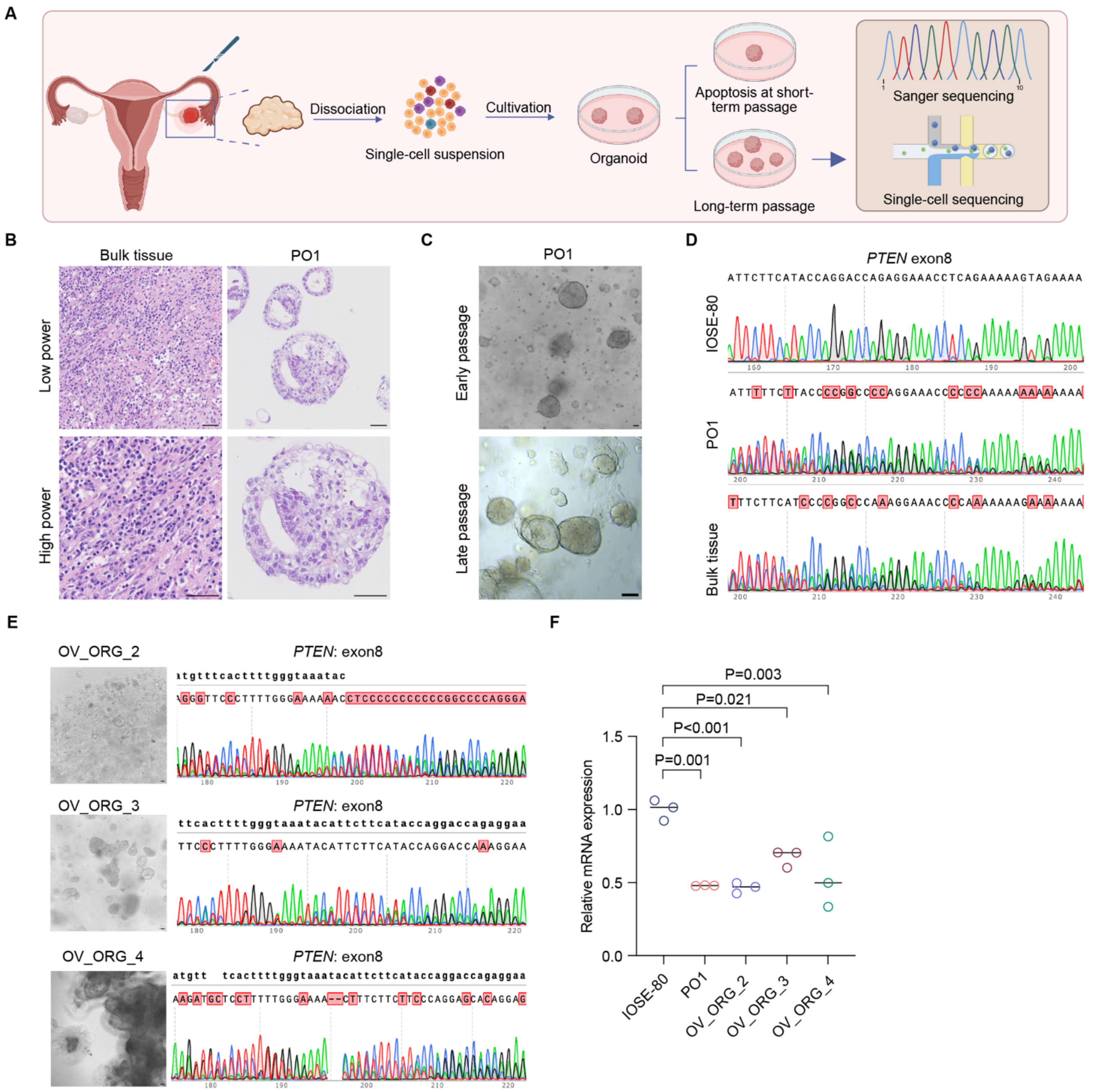

Supplementary Figure 1. *PTEN*-deleted Organoid Survived Long-term Passage

(A) Workflow schematic for organoid derivation and analyses. Fresh surgical tissue was mechanically and enzymatically dissociated, epithelial fragments were embedded in Matrigel domes and cultured in defined organoid medium, followed by passaging, sanger sequencing, and single-cell RNA sequencing. Created with BioRender.com.

(B) H&E staining of patient bulk tumor and the matched PO1 organoid. Scale bar, 50 μm.

(C) Bright-field images of PO1 organoids at early passage (P2–P3) and late passage (≥P10), showing minimal structural changes over time. Scale bar, 50μm.

(D) Sanger sequencing of *PTEN* exon 8 in IOSE-80 (immortalized ovarian surface epithelial cell line, wild-type), PO1 organoid, and matched bulk tumor. Amplicons were generated with primers flanking *PTEN* exon 8 (sequences in Methods).

(E) Additional *PTEN*-mutant organoids that persisted to passage 2 but failed to expand beyond passage 4. Left, bright-field image; right, Sanger results confirming exon-8 indels.

(F) RT–qPCR quantification of *PTEN* mRNA in *PTEN*-mutant organoids relative to IOSE-80, relative to ACTB (n = 3 independent experiments).

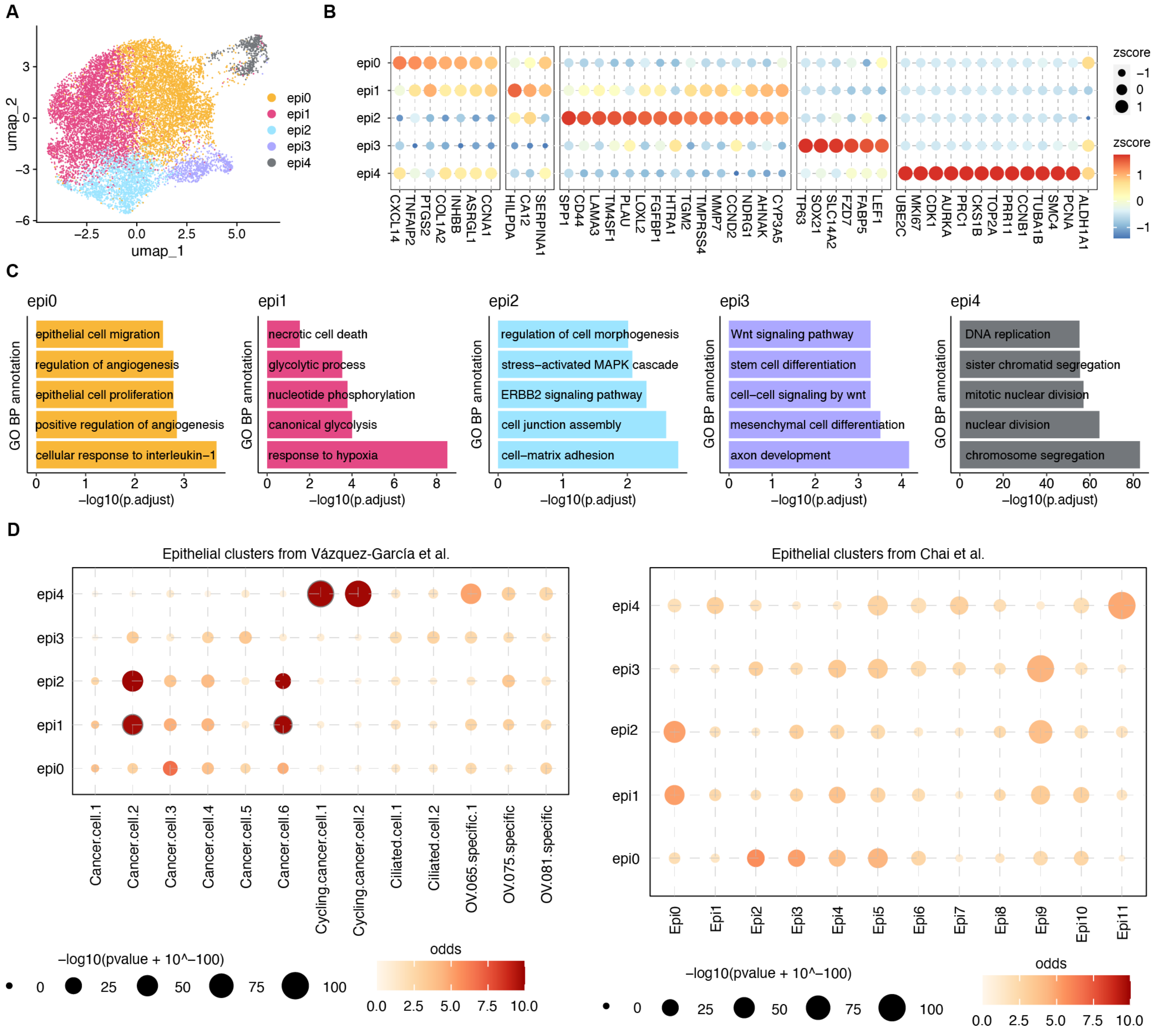

Supplementary Figure 2. scRNA-seq of the *PTEN*-deleted Organoid (PO1)

(A) UMAP plot depicting scRNA-seq data from PO1, showing distinct clusters of tumor cells.

(B) Heatmap of marker genes selected by entropy test across PO1 clusters.

(C) Gene Ontology (GO) over-representation analysis of top markers from the 5 tumor subclusters.

(D) Cross-dataset comparison of PO1 tumor programs with published human ovarian cancer single-cell references using a hypergeometric overlap test [1,2]. For each PO1 subcluster, the overlap between its marker genes and reference signatures was evaluated.

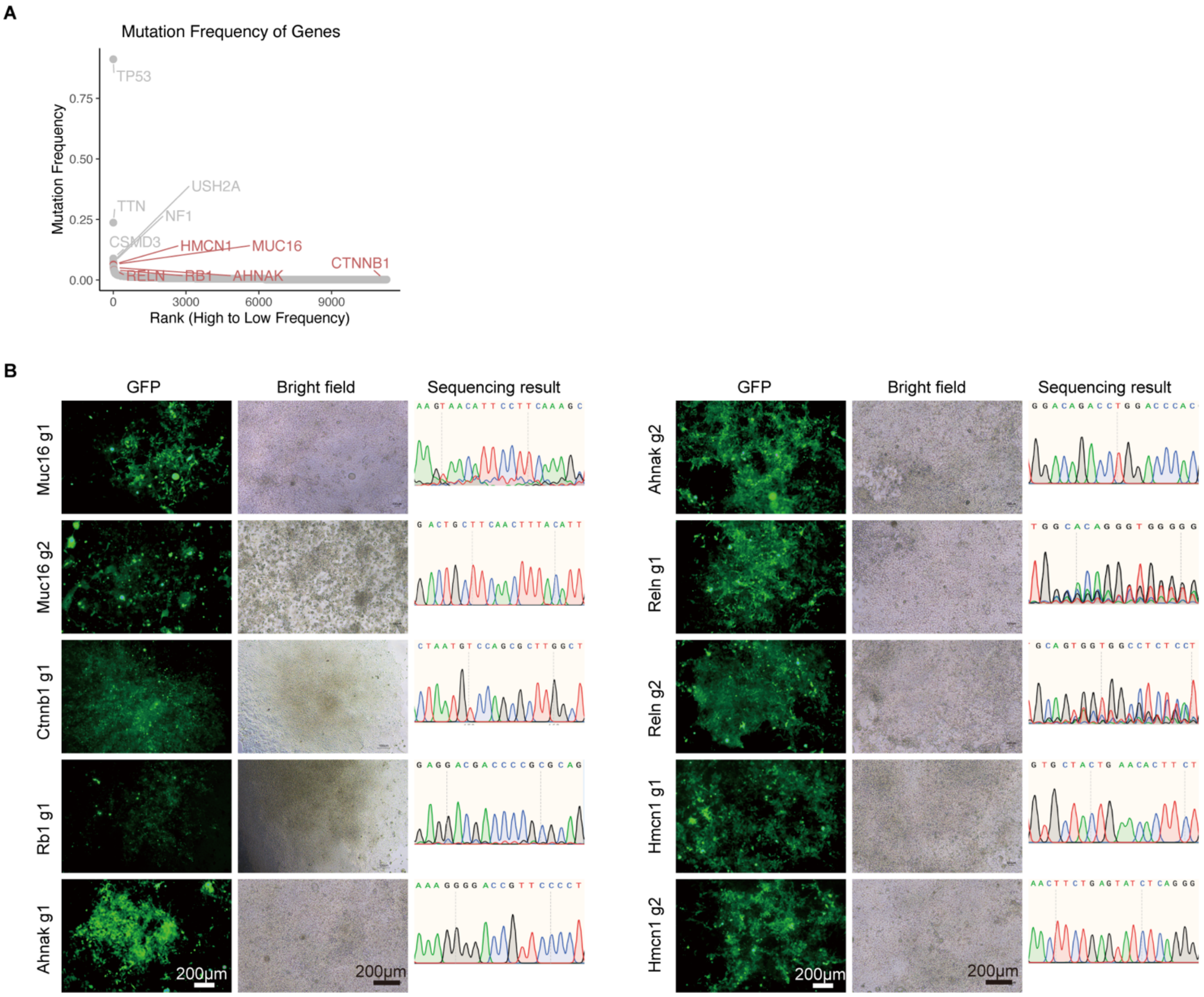

Supplementary Figure 3. Development and Optimization of the EPI-SauriCas9 System

(A) sgRNA screening for commonly mutated genes in ovarian cancer.

(B) Double-knockout EPI-SauriCas9 plasmids were constructed using the selected available sgRNA and the identified effective sgRNA for *Trp53*. These plasmids were then transfected into MOSE cells to assess whether the surviving cells could sustain long-term propagation.

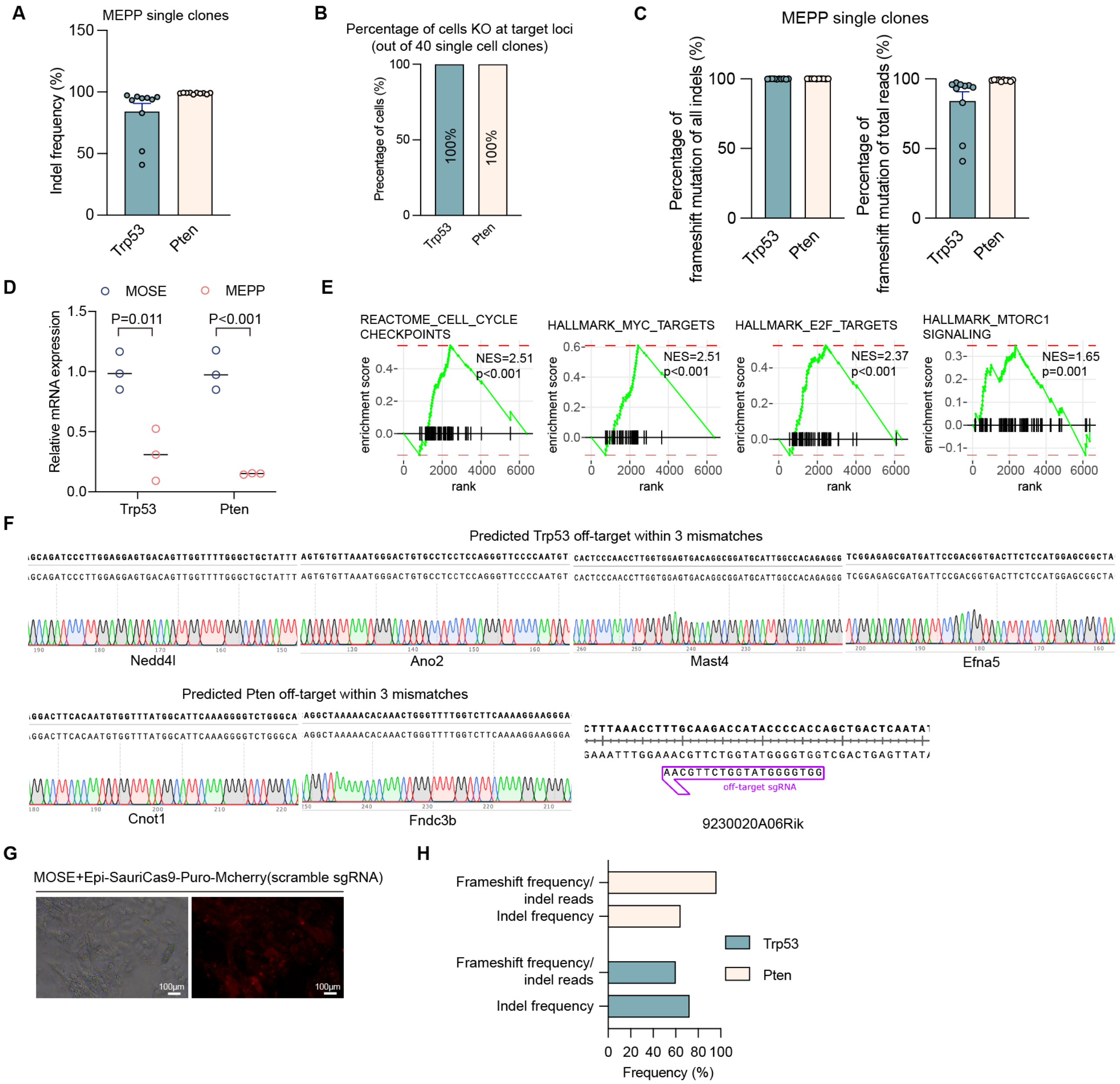

Supplementary Figure 4. EPI-SauriCas9 Mediated *Pten* and *Trp53*-Deleted Mouse Ovarian Cancer Model

(A) Amplicon deep sequencing results from 10 independently derived MEPP single-cell clones showing editing outcomes at the *Trp53* and *Pten* loci.

(B) Proportion of clones with disruption at both loci; 40/40 single-cell clones harbored indels at *Trp53* and *Pten*.

(C) Frameshift burden across 10 independently derived MEPP single-cell clones: Frameshift events accounted for ~100% of the detected indels, indicating functional disruption of both genes.

(D) RT–qPCR quantification of *Trp53* and *Pten* mRNA relative to *Gapdh*; statistical evaluation by multiple unpaired t-tests (n = 3 independent experiments).

(E) Gene-set enrichment analysis comparing MEPP with MOSE using Reactome and HALLMARK collections.

(F) Amplicon sequencing of predicted off-target sites for sgTrp53 and sgPten, confirming the absence of detectable off-target mutations.

(G) Representative bright-field and fluorescence micrographs of MOSE cells transfected with a scramble control plasmid.

(H) Amplicon sequencing of MOSE cells infected with sgRNA pair1 by lentivirus. Result showed indel frequency and frameshift frequency of *Trp53* and *Pten*, respectively.

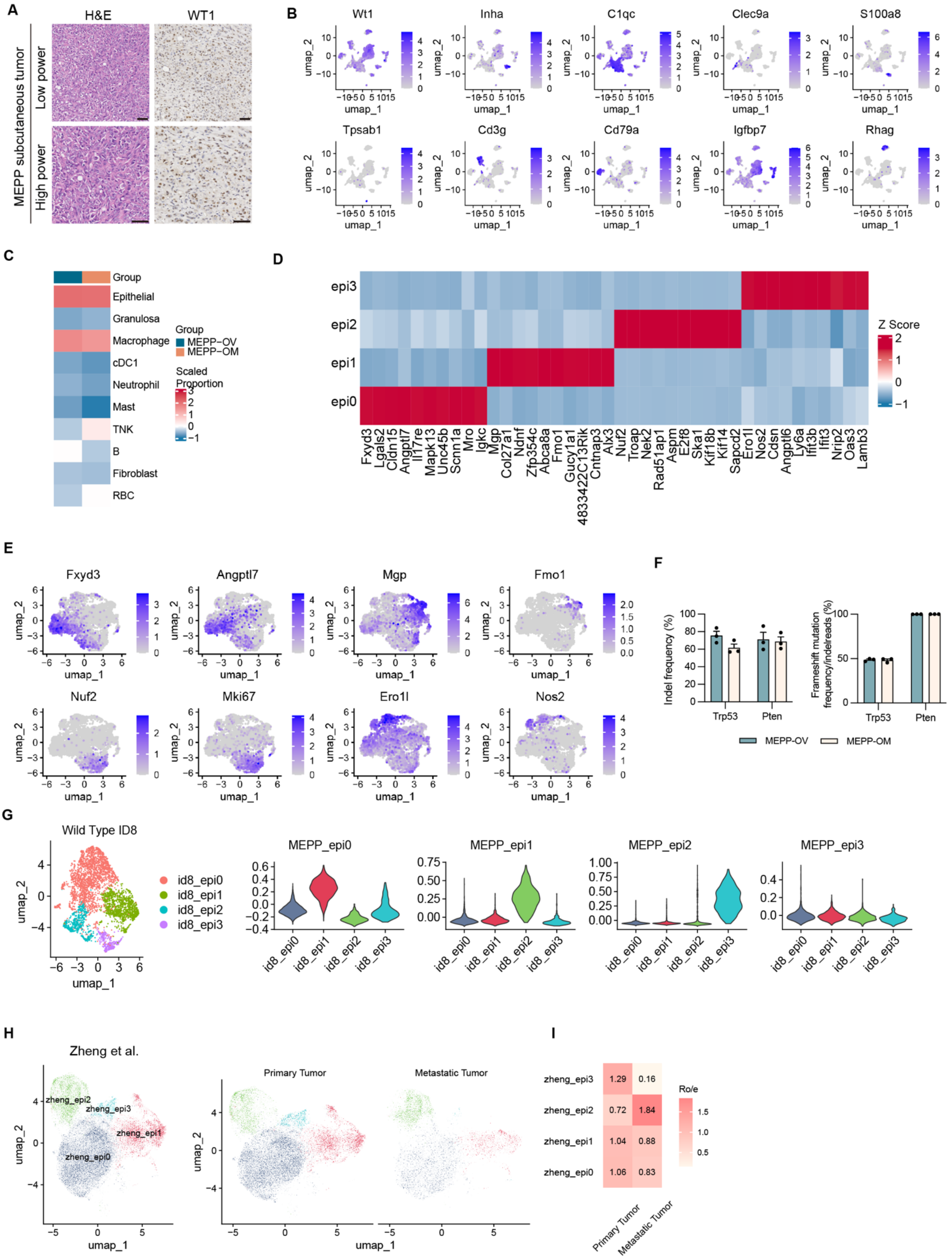

Supplementary Figure 5. scRNA-seq of MEPP Tumor

(A) Representative H&E and IHC images (WT1) of MEPP-derived subcutaneous tumors. Scale bar, 50 μm.

(B) UMAP plots showing expression of canonical lineage and tumor markers in single cells from both primary ovarian tumors and matched omental metastases.

(C) Heatmap summarizing the relative proportions of major tumor subclusters in the primary site versus omental metastases.

(D) Heatmap of the top 10 marker genes defining each tumor subcluster identified in MEPP tumors.

(E) UMAP visualization of selected representative marker genes highlighting distinct tumor subclusters.

(F) On-target indel frequencies at *Trp53* and *Pten* in primary lesions versus omental metastases (left panel). Rates were comparable between sites. Proportions of frameshifting alleles at Trp53 and Pten in MEPP-OV and MEPP-OM. Frameshift fractions were similar across sites (right panel) (n = 3 biologically independent samples).

(G) UMAP plot of ID8-wt tumor cells and violin plots depicting expression of MEPP-defining marker genes across ID8 tumor subclusters.

(H) Cross-reference with public data: UMAP plots of tumor subclusters from primary and metastatic ovarian cancer samples reported by Zheng et al. [3]

(I) Tissue distribution of MEPP tumor subclusters represented as the ratio of observed to expected cell numbers (Ro/e) in primary versus metastatic sites.

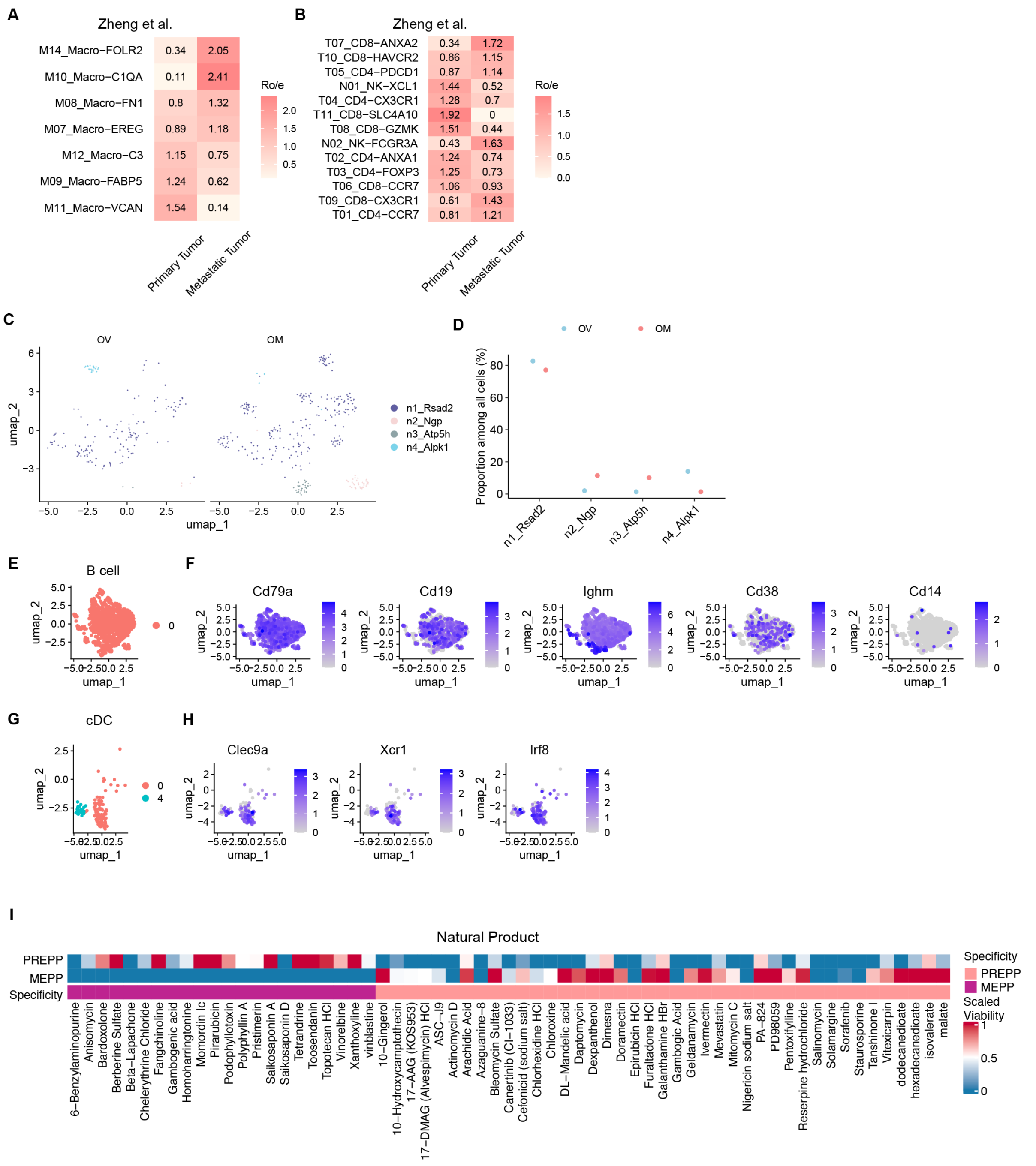

Supplementary Figure 6. Immune landscape characterization of additional immune lineages in the MEPP tumor microenvironment.

(A–B) Tissue distribution of macrophage (A) and T/NK (B) subclusters estimated by the ratio of observed to expected cell numbers (Ro/e) in primary ovarian (OV) and omental metastatic (OM) tumors in human ovarian cancer microenvironment.

(C) UMAP plot showing neutrophil clusters in OV and OM tumors of MEPP model.

(D) Dot plot depicting proportional changes of neutrophil subclusters between OV and OM tumors of MEPP model.

(E) UMAP plot identifying tumor-infiltrating B cells.

(F) UMAP plots showing expression of canonical B cell marker genes.

(G) UMAP plot identifying tumor-infiltrating conventional dendritic cells (cDCs).

(H) UMAP plots showing expression of representative cDC1 marker genes.

(I) Comparative drug sensitivity analysis of MEPP versus PREPP cells, identifying compounds preferentially targeting the *Trp53*/*Pten*-deficient background.

References

1 Vázquez-García, I. *et al.* Ovarian cancer mutational processes drive site-specific immune evasion. *Nature* **612**, 778–786 (2022).

2 Chai, C. *et al.* Single-cell transcriptome analysis of epithelial, immune, and stromal signatures and interactions in human ovarian cancer. *Commun. Biol.* **7**, 131 (2024).

3 Zheng, X. *et al.* Single-cell analyses implicate ascites in remodeling the ecosystems of primary and metastatic tumors in ovarian cancer. *Nat. Cancer* **4**, 1138–1156 (2023).
