# Supplementary material for: EPI-SauriCas9-based mouse ovarian cancer models recapitulating pten deletion in patients
Source: Commun Biol. 2025 Dec 29;9:159. doi: 10.1038/s42003-025-09437-2 (PMC12873370; doi:10.1038/s42003-025-09437-2)
Supplement: Supplementary file 3 — Description of Additional Supplementary Files [file 42003_2025_9437_MOESM3_ESM.pdf]

Description of Additional Supplementary File

File name: Supplementary Data 1

Description: Metadata of the patient organoids

File name: Supplementary Data 2

Description: scRNA-seq markers

File name: Supplementary Data 3

Description: Epithelial cell markers

File name: Supplementary Data 4

Description: Enrichment result of the epithelial subset

File name: Supplementary Data 5

Description: Gene set enrichment analysis of the human ovarian cancer dataset

File name: Supplementary Data 6

Description: The first-round drug screening result

File name: Supplementary Data 7

Description: The second-round drug screening result

File name: Supplementary Data 8

Description: Target sequence of the sgRNAs

File name: Supplementary Data 9

Description: Structural variants identified in WGS
