# Supplementary material for: EPI-SauriCas9-based mouse ovarian cancer models recapitulating pten deletion in patients
Source: Commun Biol. 2025 Dec 29;9:159. doi: 10.1038/s42003-025-09437-2 (PMC12873370; doi:10.1038/s42003-025-09437-2)
Supplement: Supplementary file 5 — Reporting summary [file 42003_2025_9437_MOESM5_ESM.pdf]

nature portfolio

Corresponding author(s): Bin Yu

Last updated by author(s): Nov 28, 2025

Reporting Summary

Nature Portfolio wishes to improve the reproducibility of the work that we publish. This form provides structure for consistency and transparency in reporting. For further information on Nature Portfolio policies, see our Editorial Policies and the Editorial Policy Checklist.

Statistics

For all statistical analyses, confirm that the following items are present in the figure legend, table legend, main text, or Methods section.

n/a	Confirmed	
☐	☒	The exact sample size (*n*) for each experimental group/condition, given as a discrete number and unit of measurement
☐	☒	A statement on whether measurements were taken from distinct samples or whether the same sample was measured repeatedly
☐	☒	The statistical test(s) used AND whether they are one- or two-sided<br>*Only common tests should be described solely by name; describe more complex techniques in the Methods section.*
☐	☒	A description of all covariates tested
☐	☒	A description of any assumptions or corrections, such as tests of normality and adjustment for multiple comparisons
☐	☒	A full description of the statistical parameters including central tendency (e.g. means) or other basic estimates (e.g. regression coefficient) AND variation (e.g. standard deviation) or associated estimates of uncertainty (e.g. confidence intervals)
☐	☒	For null hypothesis testing, the test statistic (e.g. *F*, *t*, *r*) with confidence intervals, effect sizes, degrees of freedom and *P* value noted<br>*Give P values as exact values whenever suitable.*
☐	☒	For Bayesian analysis, information on the choice of priors and Markov chain Monte Carlo settings
☐	☒	For hierarchical and complex designs, identification of the appropriate level for tests and full reporting of outcomes
☐	☒	Estimates of effect sizes (e.g. Cohen's *d*, Pearson's *r*), indicating how they were calculated

Our web collection on statistics for biologists contains articles on many of the points above.

Software and code

Policy information about availability of computer code

Data collection	No software code was used to collect the raw experimental data; all data acquisition was performed using the manufacturer supplied instrument control software and standard protocols for each platform.
Data analysis	Raw single cell RNA seq reads were adapter trimmed and quality filtered with fastp (v0.21.0) and then aligned and UMI quantified using STARsolo (v2.7.10a). Downstream analyses were conducted in R (v4.1.0) and Python (v3.6.1). Single cell clustering, marker identification, and module scoring were performed using Seurat; gene ontology enrichment was carried out with clusterProfiler (v4.2.2); pathway activity scoring employed GSVA; overlap and hypergeometric tests used GeneOverlap (v1.30.0); and gene set enrichment analysis was done with fgsea. Statistical tests (two sided Student's t test, Wilcoxon, one way and two way ANOVA) and publication quality figures were generated in R(v4.1.0) and GraphPad Prism (v10.0). No additional custom code beyond standard package functions was used.

For manuscripts utilizing custom algorithms or software that are central to the research but not yet described in published literature, software must be made available to editors and reviewers. We strongly encourage code deposition in a community repository (e.g. GitHub). See the Nature Portfolio guidelines for submitting code & software for further information.

Data

Policy information about availability of data

All manuscripts must include a data availability statement. This statement should provide the following information, where applicable:

- Accession codes, unique identifiers, or web links for publicly available datasets
- A description of any restrictions on data availability
- For clinical datasets or third party data, please ensure that the statement adheres to our policy

The raw sequence data reported in this paper have been deposited in the Genome Sequence Archive91 in National Genomics Data Center, China National Center for Bioinformation / Beijing Institute of Genomics, Chinese Academy of Sciences (GSA: CRA024029; CRA029112; CRA034026; HRA013088) that are publicly accessible at https://ngdc.cncb.ac.cn/gsa. For public datasets used in this study, scRNA-seq data from Zheng et al. are available in Mendeley Data (https://doi.org/10.17632/rc47y6m9mp.1). scRNA-seq data of wild type ID8 are available at GSE183368 94. Source data of the figures are available on figshare (https://doi.org/10.6084/m9.figshare.30691916). All other data are available from the corresponding author on reasonable request.

Research involving human participants, their data, or biological material

Policy information about studies with human participants or human data. See also policy information about sex, gender (identity/presentation), and sexual orientation and race, ethnicity and racism.

Reporting on sex and gender	All human participants whose tumor tissues were collected were biologically female; gender identity beyond biological sex was not recorded. No sex or gender based subgroup analyses were performed because only one sex was included.
Reporting on race, ethnicity, or other socially relevant groupings	All patients were of self identified Han Chinese ethnicity, as they were recruited at a single tertiary center in Shanghai. Ethnicity was recorded in the hospital medical records and was not used as a proxy for socioeconomic status; no additional race- or ethnicity based analyses were performed.
Population characteristics	Tumor specimens were obtained from adult patients diagnosed with epithelial ovarian cancer at Renji Hospital, School of Medicine, Shanghai Jiao Tong University.
Recruitment	Patients undergoing primary surgical resection for ovarian cancer at Renji Hospital were approached pre operatively and invited to donate excess tumor tissue, provided they met inclusion criteria and gave informed consent.
Ethics oversight	The collection and use of human tumor samples were approved by the Ethics Committee of Renji Hospital, School of Medicine, Shanghai Jiao Tong University.

Note that full information on the approval of the study protocol must also be provided in the manuscript.

Field-specific reporting

Please select the one below that is the best fit for your research. If you are not sure, read the appropriate sections before making your selection.

☒ Life sciences ☐ Behavioural & social sciences ☐ Ecological, evolutionary & environmental sciences

For a reference copy of the document with all sections, see nature.com/documents/nr-reporting-summary-flat.pdf

Life sciences study design

All studies must disclose on these points even when the disclosure is negative.

Sample size	No formal a priori power calculation was performed. Instead, group sizes were chosen based on our own pilot data and prior published studies showing clear differences in tumor growth and drug response with 6–8 mice per cohort for subcutaneous and orthotopic MEPP models. In vitro assays (CCK 8 viability, RT qPCR) were conducted with at least three independent biological replicates, each measured in technical triplicate.
Data exclusions	No data were excluded from any analyses. All tumor volume measurements, image based proliferation/apoptosis indices, and sequencing reads that passed standard quality control filters were included in the final datasets.
Replication	The number of biological replicates for each experiment is reported in the figure legends. Single cell RNA-seq analysis were processed using established workflows and quality control criteria as described in the Methods.
Randomization	Mice were randomly allocated to treatment groups (vehicle, FK228, thioguanine) once tumors reached 50–100mm³.
Blinding	Immunostaining images were quantified by a second investigator who was not aware of treatment assignments.

Reporting for specific materials, systems and methods

We require information from authors about some types of materials, experimental systems and methods used in many studies. Here, indicate whether each material, system or method listed is relevant to your study. If you are not sure if a list item applies to your research, read the appropriate section before selecting a response.

Materials & experimental systems

n/a	Involved in the study
☐	☒ Antibodies
☐	☒ Eukaryotic cell lines
☒	☐ Palaeontology and archaeology
☐	☒ Animals and other organisms
☒	☐ Clinical data
☒	☐ Dual use research of concern
☒	☐ Plants

Methods

n/a	Involved in the study
☒	☐ ChIP-seq
☒	☐ Flow cytometry
☒	☐ MRI-based neuroimaging

Antibodies

Antibodies used	WT1 (1:200 ;abcam, Cat# ab89901), Ki67 (1:200; abcam, Cat# ab16667), N-cadherin (1:200; Proteintech, Cat# 22018-1-AP), p-ERK1/2 (1:100; CST, Cat# 9101), and ERα (1:100; abcam, Cat# ab32063), HRP-conjugated secondary anitbody (1:200; abcam, Cat# ab6721)
Validation	Each antibody has been validated for immunohistochemistry on mouse/human tissue.

Eukaryotic cell lines

Policy information about cell lines and Sex and Gender in Research

Cell line source(s)	Cell lines used in the study included ID8 mouse ovarian surface epithelial cells (mouse; MERCK, Cat# SCC145), Hepa1-6 (mouse; ATCC, Cat# CRL-1830), A2780 (human; Cell Bank, Chinese Academy of Sciences, Cat# SCSP-5477), COV362 (human; QuiCell Biotechnology, Cat# C772), HEY (human; QuiCell Biotechnology, Cat# H1808), OVCAR3 (human; ATCC, Cat# HTB-161), OVCAR8 (human 80), SKOV3 (human; ATCC, Cat# HTB-77) and IOSE-80 (QuiCell Biotechnology, Cat# I421).
Authentication	Cell lines were authenticated by STR profiling.
Mycoplasma contamination	Cell lines were tested negative for mycoplasma contamination.
Commonly misidentified lines (See ICLAC register)	NA

Animals and other research organisms

Policy information about studies involving animals; ARRIVE guidelines recommended for reporting animal research, and Sex and Gender in Research

Laboratory animals	Mouse line used in this study is C57BL/6JGpt unless otherwise stated (hereafter referred to as C57BL/6; GemPharmatech Co., Ltd, Strain NO. N000013; MGI identifier, 6314664). NOD/ShiLtJGpt-Prkdcem26Cd52Il2rgem26Cd22/Gpt (hereafter referred to as NCG; GemPharmatech Co., Ltd, Strain NO. T001475) was used for patient-derived xenograft (PDX) experiments.
Wild animals	NA
Reporting on sex	All laboratory animals were female.
Field-collected samples	NA
Ethics oversight	The experiments were approved by the Ethics Committee of Renji Hospital, School of Medicine, Shanghai Jiao Tong University.

Note that full information on the approval of the study protocol must also be provided in the manuscript.

Plants

Seed stocks	NA
Novel plant genotypes	NA
Authentication	NA
